# Supplementary material for: Exploring the Role of the Environment as a Reservoir of Antimicrobial-Resistant Campylobacter: Insights from Wild Birds and Surface Waters
Source: Microorganisms. 2024 Aug 8;12(8):1621. doi: 10.3390/microorganisms12081621 (PMC11356556; doi:10.3390/microorganisms12081621)
Supplement: Supplementary file 1 [file microorganisms-12-01621-s001.zip › Table S1.pdf]

***bla**$_{OXA}$* **genes**	**No. of isolates**	**Proportion out of 126 isolates with *bla**$_{OXA}$* genes**	**No. of resistant phenotype**
bla$_{OXA-193}$	33 including 4 *C. coli*	26.2% (CI95: 19.3 – 34.5%)	2
bla$_{OXA-184}$	30	23.8% (CI95: 17.2 – 32%)	2
bla$_{OXA-447}$	17	13.5% (CI95: 8.6 – 20.5%)	
bla$_{OXA-61}$	16 including 15 *C. coli*	12.7% (CI95: 8.0 – 19.6%)	1
bla$_{OXA-638}$	7	5.6% (CI95: 2.7 – 11%)	1
bla$_{OXA-637}$	3	2.4% (CI95: 0.8 – 6.8%)	0
bla$_{OXA-446}$	3	2.4% (CI95: 0.8 – 6.8%)	0
bla$_{OXA-617}$	3	2.4% (CI95: 0.8 – 6.8%)	0
bla$_{OXA-631}$	2	1.6% (CI95: 0.4 – 5.6%)	0
bla$_{OXA-639}$	2	1.6% (CI95: 0.4 – 5.6%)	0
bla$_{OXA-785}$	2	1.6% (CI95: 0.4 – 5.6%)	0
bla$_{OXA-622}$	1	0.8% (CI95: 0.1 – 4.4%)	0
bla$_{OXA-597}$	1	0.8% (CI95: 0.1 – 4.4%)	0
bla$_{OXA-616}$	1	0.8% (CI95: 0.1 – 4.4%)	0
bla$_{OXA-624}$	1	0.8% (CI95: 0.1 – 4.4%)	0
bla$_{OXA-658}$	1	0.8% (CI95: 0.1 – 4.4%)	0
bla$_{OXA-452}$	1	0.8% (CI95: 0.1 – 4.4%)	0
bla$_{OXA-466}$	1	0.8% (CI95: 0.1 – 4.4%)	0
bla$_{OXA-615}$	1	0.8% (CI95: 0.1 – 4.4%)	0
Susceptible	137		3
