# Supplementary figures and images for: Exploring the Role of the Environment as a Reservoir of Antimicrobial-Resistant Campylobacter: Insights from Wild Birds and Surface Waters
Source: Microorganisms. 2024 Aug 8;12(8):1621. doi: 10.3390/microorganisms12081621 (PMC11356556; doi:10.3390/microorganisms12081621)

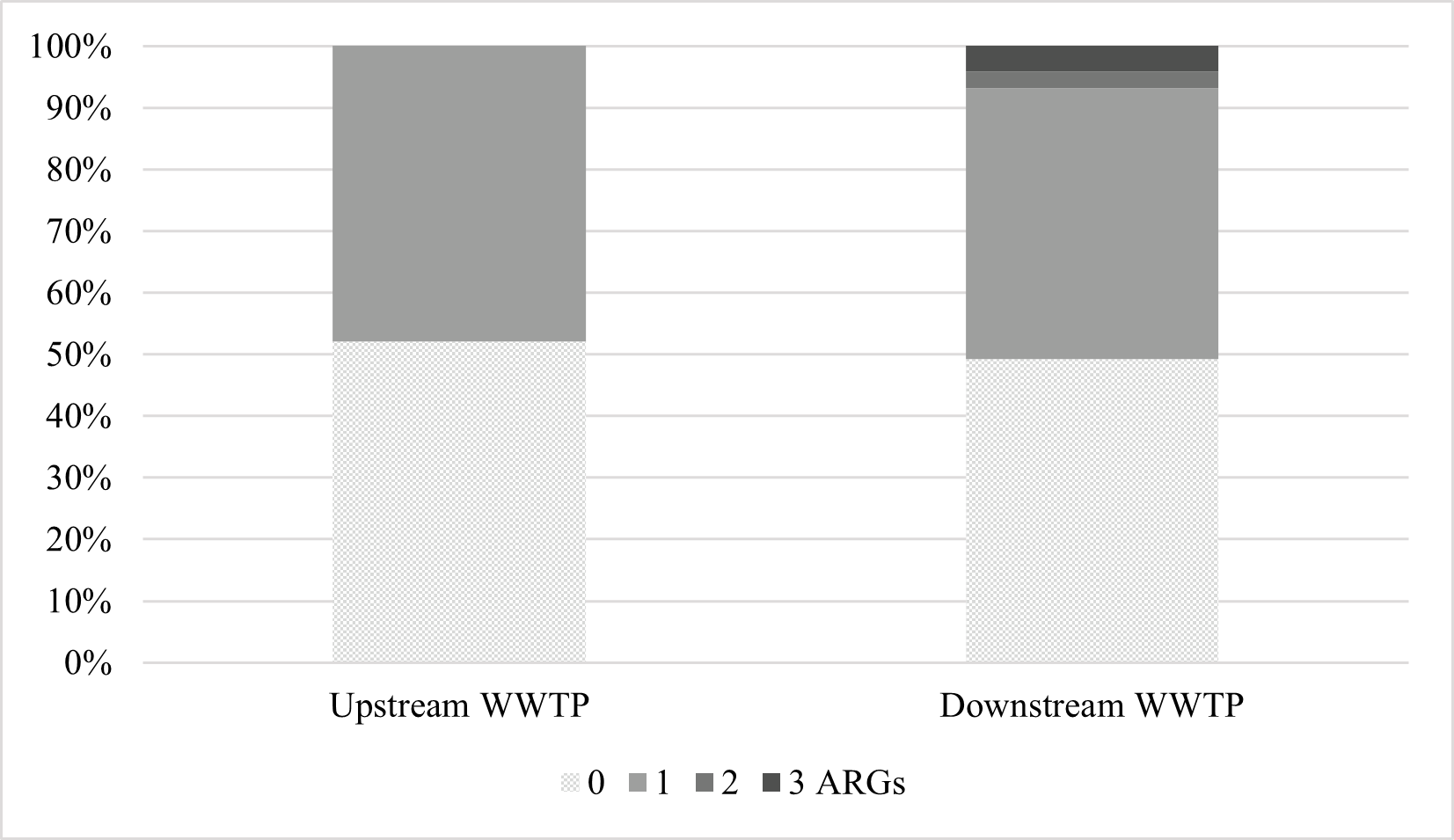

Supplement: Supplementary file 1 [file microorganisms-12-01621-s001.zip › Figure S1.jpg]

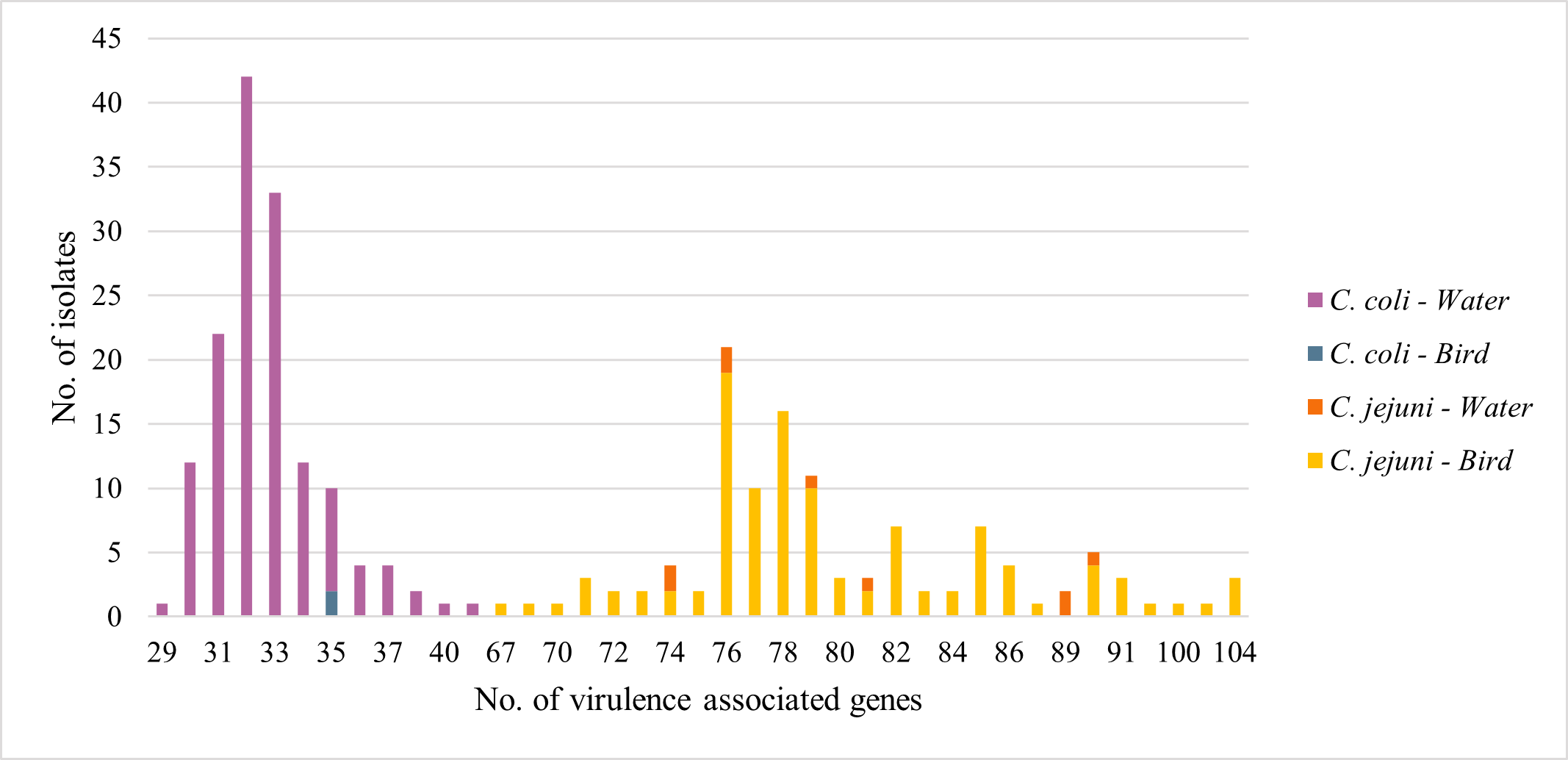

Supplement: Supplementary file 1 [file microorganisms-12-01621-s001.zip › Figure S2.jpg]
